# Supplementary material for: DExD/H Box Helicases DDX24 and DDX49 Inhibit Reactivation of Kaposi’s Sarcoma Associated Herpesvirus by Interacting with Viral mRNAs
Source: Viruses. 2022 Sep 20;14(10):2083. doi: 10.3390/v14102083 (PMC9609691; doi:10.3390/v14102083)
Supplement: Supplementary file 1 [file viruses-14-02083-s001.zip › Figures S1-S3.pdf]

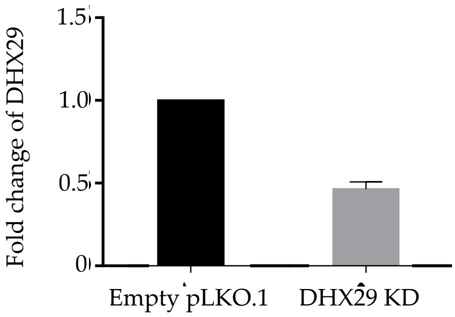

Figure S1. Validation of Stably Transduced DHX29 knockdown (KD) BCBL-1 cells. Lentivirus transduced BCBL-1 cells express 5 shRNAs to target DHX29. DHX29 transcript abundance from DHX29 KD is shown relative to the empty vector control. n=3.

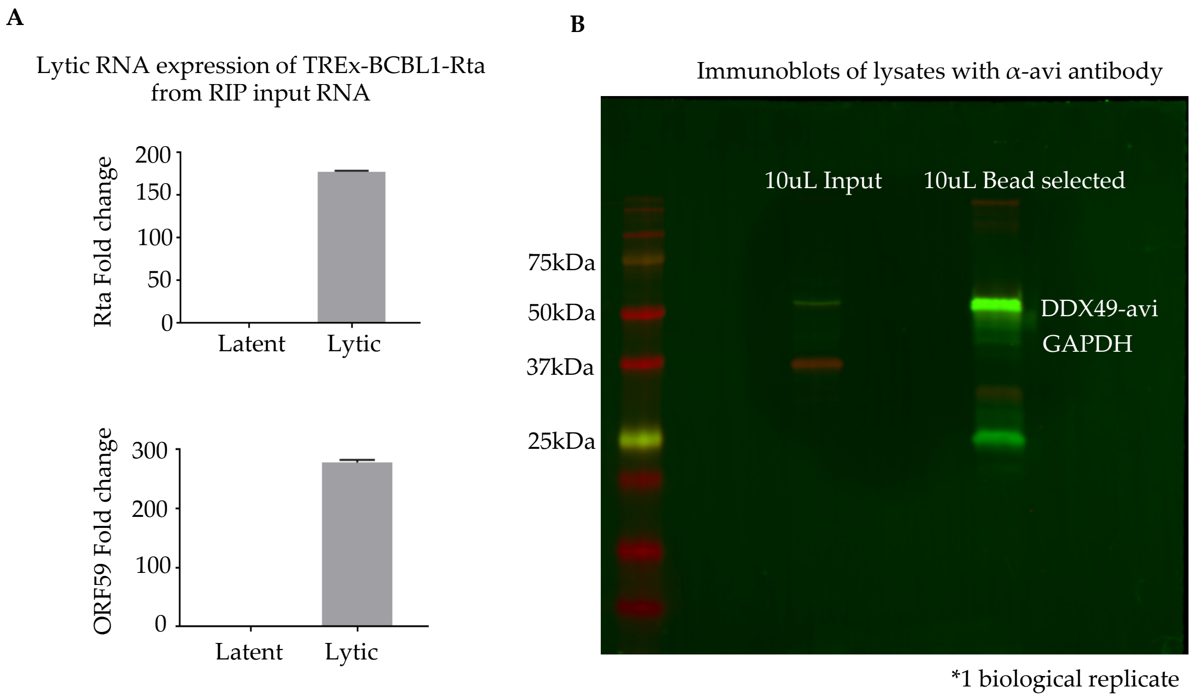

Figure S2. Validation of TREx BCBL1-Rta reactivation and immunoprecipitation conditions for RNA Immunoprecipitation analysis. (**A**) qPCR of TREx BCBL1-Rta cells during latency or induced for 24 hours with 1 ug/mL Doxycycline and 0.25 mM PFA. The left top figure represents the relative fold change in expression of the immediate early Rta gene normalized with GAPDH. The figure below represents early ORF59 expression. (**B**) Immunoblot of lysates prior to pulldown (input) and after being pulled down with an α-Avidin antibody. 10 uL of lysate was run on a Mini-PROTEAN TGX gel, Biorad.

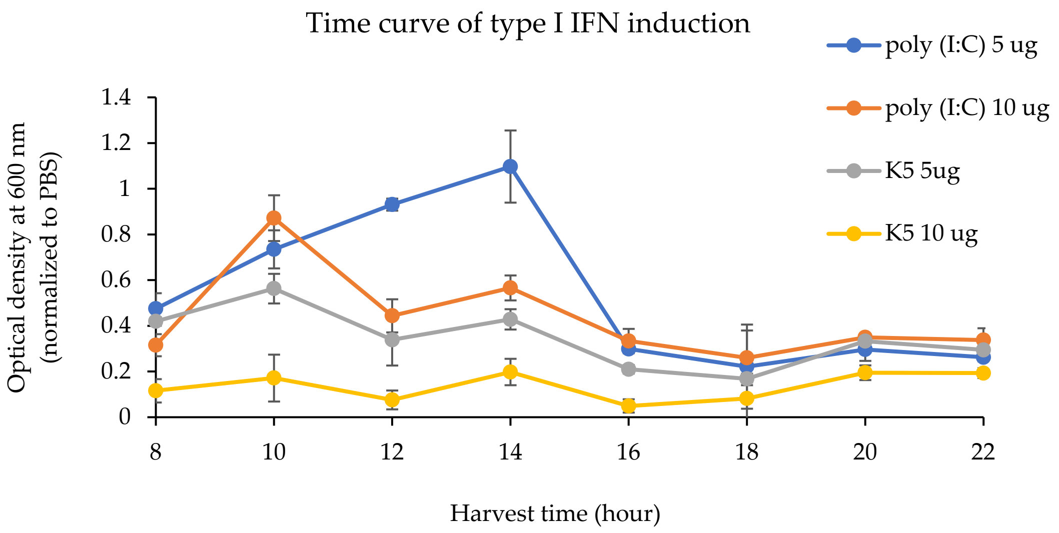

Figure S3. Time curve of type I IFN induction detected by HEK-blue IFNα/β™ cells transfected with either 5 ug and 10 ug poly(I:C) or 5 ug and 10 ug of pcDNA3.1 plasmid expressing KSHV gene, K5. K5, KSHV lytic transcript that was identified in RIP; poly (I:C) as positive control. n=3.
