# Supplementary material for: DExD/H Box Helicases DDX24 and DDX49 Inhibit Reactivation of Kaposi’s Sarcoma Associated Herpesvirus by Interacting with Viral mRNAs
Source: Viruses. 2022 Sep 20;14(10):2083. doi: 10.3390/v14102083 (PMC9609691; doi:10.3390/v14102083)
Supplement: Supplementary file 1 [file viruses-14-02083-s001.zip › Tables S1-S5.pdf]

Table S1. The 22 DExD/H box helicase targets of the knockdown screen.

Target DExD/H box gene
EIF4A1
EIF4A2
DDX3X
DDX5
DDX11
DDX18
DDX21
DDX24
DHX29
DDX31
DHX36
DHX37
DHX40
DDX41
EIF4A3
DDX49
DDX52
DDX55
DHX58
DHX9
DDX58 (RIG-I)
MDA5 (RLR)

[siRNA pools used were a gracious gift from the McFadden lab. siRNA screen of 22 DExD/H box helicases in Vero cells infected with rKSHV.219. All transient KD Vero cells looked healthy 28 hours after siRNA transfection and prior to chemical induction, with negligible spontaneous background reactivation.]

Table S2. Pooled siRNAs used in the Vero knockdown screen (ON-TARGETplus Dharmacon)

**Dharmacon siRNA pools used in Vero Screen**	
**Target**	**Sequence**
DDX24	5′-GCAAGAAGAACGUCGGAGA-3′
L-010397-01-0005*	5′-AGACAAAGAAGCCGAAGGA-3′
	5′-CCGUUUAGCUCGACAGAUU-3′
	5′-GAACCGUCGUCCUGAGAUU-3′
DHX29	5′-CUGCAGAUCAUUACGGAAU-3′
L-013759-01-0005*	5′-GAGUGAUCAAUGAGCAUAA-3′
	5′-CGAAGAAUCUCAGCAGUUA-3′
	5′-AAAUGUAGGGUUAGGGUAA-3′
DDX49	5′-CGGCCCACUUUGACGAAAA-3′
L-017975-01-0005*	5′-AGGUCAACGUGGUGCGAAG-3′
	5′-GUAUAAAGAAGAUCCGCUU-3′
	5′-CUGAGAAGGUCAAGGACGC-3′
Non-targeting Pool	5′-UGGUUUACAUGUCGACUAA-3′
D-001810-10-05*	5′-UGGUUUACAUGUUGUGUA-3′
	5′-UGGUUUACAUGUUUUCUGA-3′
	5′-UGGUUUACAUGUUUUCCUA-3′

*Dharmacon catalog number

Table S3. Sequences of PCR primers.

Target	Sequence[a]
Human GAPDH	F: 5'-CTTTGGTATCGTGGAAGGACTC-3'
Human GAPDH	R: 5'-GTAGAGGCAGGGATGATGTTC-3'
RTA	F: 5'-CACAAAAATGGCGCAAGATGA-3'
RTA	R: 5'-TGGTAGAGTTGGGCCTTCAGTT-3'
LANA	F: 5'-GCGCCCTTAACGAGAGGAAGTT-3'
LANA	R: 5'-TTCCTTCGCGGTTGTAGATG-3'
K8.1	F: 5'-CCACCACAGAACTGACCGAT-3'
K8.1	R: 5'-GTGGTGGCAGAAAATGGCAC-3'
ORF59	F: 5'-TTAGAAGTGGAAGGTGTGCC-3'
ORF59	R: 5'-TCCTGGAGTCCGGTATAGAATC-3'
pCMV-DDK	F: 5'-AGAAGAGGATCTGGCAGCAAA-3'
pCMV-DDK	R: 5'-GGCCGGCCGTTTAAACCTTAT-3'
DDX24	F: 5'-GGCCAGGTTTACAGGAATTA-3'
DDX24	R: 5'-GGGTTGTATTGGGAGTCATT-3'
DHX29	F: 5'-TCTCGAGCTTGTGAATCTAC-3'
DHX29	R: 5'-CTGACTGGACACTTCTTTCA-3'
DDX49	F: 5'-TTGTCCTTCCCATCTTGCAG-3'
DDX49	R: 5'-CGACGATGATGCAGTCTTTC-3'
DDX58	F: 5'-ACCTACATCCTGAGCTACAT-3'
DDX58	R: 5'-TCATCCCCTTAGTAGAGCAA-3'
Avi	R: 5'-TGCCATTCAATTTTCTGCGCT-3'
DHX29 ORF	F: 5'-GCAGCTGAGAGTTCTCATTGAT-3'
DDX24 ORF	F: 5'-GCCCCAAGTAAGAGCGAGTC-3'
DDX49 ORF	F: 5'-CGCTTCAAGGAGAAGGTGGAG-3'
IFN-α	F: 5′-ATGGCTAGGCTCTGTGCTTTCCT-3′
IFN-α	R: 5′-AGGGCTCTC CAGACTTCTGCTCTG-3′
IFN-β	F: 5′-CCCTATGGAGATGACGGAGA-3′
IFN-β	R: 5′-TCCCACGTCAATCTT TCCTC-3′

[a] F, forward; R, reverse

Table S4. Pool of RNAi (TRC) shRNAs used to generate DHX29 KD BCBL-1 stably transduced cells.

GE Dharmacon Clone ID	Mature Antisense Sequence	Targets
TRCN0000051238	AATAGTGGCTTGTATTTGAGG	all human transcript variants of DHX29 ORF
TRCN0000051239	ATGACAGCTATCTTTACAGGG	ORF of NM_001345964.1
TRCN0000051240	ATTGTAGGGCATACTACCAGG	all human transcript variants of DHX29 ORF
TRCN0000051241	TAATTCCTCCAAAGGTACACG	all human transcript variants of DHX29 ORF
TRCN0000051242	TAGAAGTTTCTGATACTTAGG	all human transcript variants of DHX29 ORF

[This pool of shRNAs was packaged individually into lentivirus and used to transduce BCBL-1 cells at a total pooled Multiplicity of Infection (MOI) of 10.]

Table S5. pcDNA3.2 clones of KSHV genes

No.	KSHV gene	Clone ID
1	K2	11060-X09-780
2	ORF70	11060-X12-780
3	K4	11060-X13-780
4	K4.1	11060-X14-780
5	K4.2	11060-X15-780
6	K5	11060-X16-780
7	K9	11060-X62-780
8	ORF58	11060-X66-780
9	ORF59	11060-X67-780
10	ORF65	11060-X73-780
11	ORF66	11060-X74-780
12	ORF67	11060-X75-780
